# Enhanced Data Representation by Kernel Metric Learning for Dementia Diagnosis

**DOI:** 10.3389/fnins.2017.00413

**Published:** 2017-07-26

**Authors:** David Cárdenas-Peña, Diego Collazos-Huertas, German Castellanos-Dominguez

**Affiliations:** Signal Processing and Recognition Group, Universidad Nacional de Colombia Manizales, Colombia

**Keywords:** metric learning, computer-aided diagnosis, centered kernel alignment, ADNI, magnetic resonance imaging

## Abstract

Alzheimer's disease (AD) is the kind of dementia that affects the most people around the world. Therefore, an early identification supporting effective treatments is required to increase the life quality of a wide number of patients. Recently, computer-aided diagnosis tools for dementia using Magnetic Resonance Imaging scans have been successfully proposed to discriminate between patients with AD, mild cognitive impairment, and healthy controls. Most of the attention has been given to the clinical data, provided by initiatives as the ADNI, supporting reliable researches on intervention, prevention, and treatments of AD. Therefore, there is a need for improving the performance of classification machines. In this paper, we propose a kernel framework for learning metrics that enhances conventional machines and supports the diagnosis of dementia. Our framework aims at building discriminative spaces through the maximization of center kernel alignment function, aiming at improving the discrimination of the three considered neurological classes. The proposed metric learning performance is evaluated on the widely-known ADNI database using three supervised classification machines (*k*-nn, SVM and NNs) for multi-class and bi-class scenarios from structural MRIs. Specifically, from ADNI collection 286 AD patients, 379 MCI patients and 231 healthy controls are used for development and validation of our proposed metric learning framework. For the experimental validation, we split the data into two subsets: 30% of subjects used like a blindfolded assessment and 70% employed for parameter tuning. Then, in the preprocessing stage, each structural MRI scan a total of 310 morphological measurements are automatically extracted from by FreeSurfer software package and concatenated to build an input feature matrix. Obtained test performance results, show that including a supervised metric learning improves the compared baseline classifiers in both scenarios. In the multi-class scenario, we achieve the best performance (accuracy 60.1%) for pretrained 1-layered NN, and we obtain measures over 90% in the average for HC vs. AD task. From the machine learning point of view, our proposal enhances the classifier performance by building spaces with a better class separability. From the clinical application, our enhancement results in a more balanced performance in each class than the compared approaches from the CADDementia challenge by increasing the sensitivity of pathological groups and the specificity of healthy controls.

## 1. Introduction

Alzheimer's Disease (AD) corresponds to a progressive cognitive impairment and loss of memory functions, becoming the kind of dementia with the largest prevalence in elderly subjects with nearly 44 million patients worldwide. Besides, Mild Cognitive Impairment (MCI), a previous stage of AD, affects 10–20% of people aged 65 or older (Alzheimer's Association, 2016). Hence, dementia diagnosis and treatment demand reliable biomarkers providing an objective and early characterization of the different AD stages (Shi et al., 2015). Among these biomarkers, structural magnetic resonance imaging (MRI) data became frequently used to develop computer-aided diagnosis (CAD) tools due to its wide availability and non-invasiveness (Jack et al., 2013). CAD tools learn to discriminate dementia through MRI features, benefiting from large amounts of neuroimaging data.

Particularly, the Alzheimer's Disease Neuroimaging Initiative (ADNI) focuses its researches on discriminating pathologies with a variety of classification tools from neuroimaging data, genetic information, and other biomarkes. However, insufficient attention has been given to build appropriate metrics from the training data that could maximize the performance of several classifiers (Shi et al., 2015).

In this regard, learning distance metrics tuned for classification tasks from given prior information, known as *Metric Learning* (ML), implicitly transforms input features into discriminative ones. As an example, linear dimensionality reduction changes the metric in the original space to maximize an objective function (Fukumizu et al., 2004). Therefore, introducing a metric learning stage into the discrimination process can significantly improve the performance of distance-based machines as k-nn, k-means, and even SVMs (Xu et al., 2012). Depending on the transformation to be sought, ML can be divided into linear and nonlinear models.

Linear models aim at estimating an optimal affine transformation for the input space, with straightforward interpretation, optimization simplicity, and reduced overfitting (Bellet et al., 2013). Among these models, principal component analysis (PCA) finds the subspace best preserving the variance of the data. Wachinger et al. (2016) stacks PCA matrices and logistic regressors in a multi-layer architecture. However, the generative properties of the resulting machine highlight over the discriminative ones. In the same way, Khedher et al. (2015) proposes a dimension reduction approach based on performing an independent component analysis (ICA) over high-dimensional voxel-wise features. Despite improving the accuracy of an SVM classifier, the maximization of statistical independencence of ICA may eliminate relationships between the dependent features that hold discriminative information, and restricts the resulting dimension to the number of involved classes. In addition, when handling data distributions with nonlinear structures, linear models show inherently limited performance and class separation capability (Shi et al., 2015).

On the other hand, the most popular nonlinear models are built through kernel-based methods, that embed input features into higher dimensional spaces with an increased linear separability. For instance, Awate et al. (2017) develop a kernel-based framework for building cortex descriptors that detect population differences and regress clinical variables. Specifically for AD diagnosis, Zhang et al. (2011) combines three different biomarkers using a simple-while-effective multiple-kernel learning for improving the SVM-based classification of AD and MCI. However, optimization of kernel weighting is carried out by a grid search, which is very time consuming when the number of features and samples gets large (Liu et al., 2014). Another approach uses multiple-kernels, each of them representing anatomically meaningful brain regions, to improve AD discrimination (Young et al., 2015). The kernel combination weights, that are computed via Gaussian Process framework, can be clinically read. Despite enhancing the performance of linear methods for many highly nonlinear problems, these kind of kernel-based solutions are prone to over-fitting (Bellet et al., 2013), and their utilization is inherently limited by the sizes of the kernel matrices (He et al., 2013). Another kind of non-linear models combine multiple local metrics to learn a feature transformation based on either local neighborhoods or class memberships (Wang et al., 2012). Although these multi-metric strategies are usually more powerful in modeling nonlinear structures, generalizing these methods to train robust classifiers is not trivial. In addition, the estimation of high-level complexity distances on such metric manifolds also yields to high computational cost (Shi et al., 2015).

In this paper, we introduce a kernel-based metric learning framework for supporting the dementia diagnosis task. The proposed approach searches for projections into more discriminative spaces so that the resulting data distribution resembles as much as possible the label distribution. Hence, we incorporate kernel theory for assessing the affinity between projected data and available labels through the Center Kernel Alignment (CKA) criterion. Maximization of CKA produces discriminative features aimed at improving the discrimination neurological classes. Furthermore, the proposed metric learning is introduced into three commonly used supervised classification machines, namely, *k*-nearest neighbors (*k*-nn), support vector machines (SVM) and neural networks (NN). For the latter, we generalize the supervised linear projection as a multilayer architecture. Our approach is evaluated on two scenarios of dementia diagnosis from structural MRIs (multi-class and bi-class). To this end, we use morphological measurements (volume, area, and thickness) computed by the widely used FreeSurfer suite (Tustison et al., 2014). Attained test classification results of several performance measures show that introducing a supervised metric learning improves other the baseline classifiers in both considered scenarios. Therefore, the proposal builds spaces with a better class separability that increases both the sensitivity and specificity, with the additional benefit of a more balanced the performance for each class.

The agenda of this paper is organized as follows: Firstly, we describe the mathematical framework and our proposed approach in Section 2. Then, Section 3 illustrates the carried out evaluations on the well-know ADNI collection for tuning the parameters of the CKA and classifiers. Lastly, we describe all obtained results and discuss concluding remarks in Section 4 and 5, respectively.

## 2. Materials and methods

### 2.1. Centered kernel alignment

Kernel functions are bivariate measures of similarity, which are based on the inner product between samples embedded in a Hilbert space. For an input feature space X, a kernel $\kappa_{X}:X\times X\to {\mathbb{R}}^{+}$ is a positive-definite function that defines an implicit mapping $\varphi_{X}:X\to H_{X}$, aiming to embed a data point *x* ∈ X into the element φ_*X*_(*x*) ∈ H_*X*_ of some Reproducing Kernel Hilbert Space (RKHS) (noted as H_*X*_). Within a supervised learning framework, a kernel $\kappa_{L}:L\times L\to {\mathbb{R}}^{+}$ is also introduced that acts over the target space L to account for the attribute labeling information so that κ_*L*_ defines the implicit mapping φ_*L*_(*l*):L→H_*L*_. Due to each function (κ_*X*_ and κ_*L*_) reflects a different notion of similarity extracted from a distinct sample set, the concept of alignment between mappings can be introduced to measure the degree of agreement between the input and target kernels. To unify both tasks into a coherent optimization problem, we employ the Centered Kernel Alignment (CKA) that assesses the kernel affinity through the expected value of their normalized inner product over all data points as follows:

(1) $$\rho (\kappa_{X},\kappa_{L})=\frac{{𝔼}_{xx^{\prime }ll^{\prime }}\{{\overset{-}{\kappa }}_{X}(x,x^{\prime }){\overset{-}{\kappa }}_{L}(l,l^{\prime })\}}{\sqrt{{𝔼}_{xx^{\prime }}\{{\overset{-}{\kappa }}_{X}^{2}(x,x^{\prime })\}{𝔼}_{ll^{\prime }}\{{\overset{-}{\kappa }}_{L}^{2}(l,l^{\prime })\}}},$$

where notation 𝔼E_*z*_{·} stands for the expected value of the random variable *z*, ${\overset{-}{\kappa }}_{Z}\left(z,z^{\prime }\right)$ is the centered version of the kernel function $\kappa_{Z}\left(z,z^{\prime }\right)={\left(\varphi_{Z}\left(z\right)-{\overset{-}{\varphi }}_{Z}\right)}^{⊤}\left(\varphi_{Z}\left(z^{\prime }\right)-{\overset{-}{\varphi }}_{Z}\right)$, being ${\overset{-}{\varphi }}_{Z}\in H_{Z}$ the expected value of the data distribution on H_*Z*_.

In practice, the characterizing kernel matrices, $K_{X}\in {\mathbb{R}}^{N\times N}$ and $K_{L}\in {\mathbb{R}}^{N\times N}$, are extracted from a provided input dataset ***X*** ∈ ℝ^*N* × *P*^, holding samples $x_{n}\in {\mathbb{R}}^{P}$, along with its corresponding target vector $l=\left\{l_{n}\subset \mathbb{Z}:n\in \left[1,N\right]\right\}\in {\mathbb{Z}}^{N}$. Hence, the empirical estimate for the CKA value can be computed as follows:

(2) $$\hat{\rho }\left({\overset{-}{K}}_{X},{\overset{-}{K}}_{L}\right)=\frac{{\langle {\overset{-}{K}}_{X},{\overset{-}{K}}_{L}\rangle }_{F}}{\sqrt{{\langle {\overset{-}{K}}_{X},{\overset{-}{K}}_{X}\rangle }_{F}{\langle {\overset{-}{K}}_{L},{\overset{-}{K}}_{L}\rangle }_{F}}},$$

where notation 〈·, ·〉_F_ stands for the matrix-based Frobenius product, and $\overset{-}{K}\;=\;{\left(\varphi_{Z}-{\overset{-}{\varphi }}_{Z}\right)}^{⊤}\left(\varphi_{Z}-{\overset{-}{\varphi }}_{Z}\right)$ is the centered kernel matrix (associated with ${\overset{-}{\kappa }}_{Z}\left(,\right)$) computed as $\overset{-}{K}=\overset{-}{I}K\overset{-}{I}$, being *1* ∈ ℝ^*N* × 1^ the all-ones vector, *I* the identity matrix, and $\overset{-}{I}=\left[I-\;{\mathrm{11}}^{⊤}/N\right]$.

The inner product of both kernel functions in Equation (2) estimates the agreement between the data jointly sampled from the spaces X and L through their statistical dependence ρ ∈ [0, 1]. Therefore, the larger the value of the CKA, the more similar the distributions of the input and target data. As a result, using ρ as the cost function to maximize the dependence between the characterizing kernels of the input data and the class label promotes the class discrimination from the given input samples (Gretton et al., 2005).

### 2.2. Supervised metric learning for classification

The CKA dependence using the Mahalanobis metric learning is developed for three commonly used approaches to supervised classification: *k*-nn and SVM that are fed into a Gaussian kernel optimization, and NN that are optimized by a backpropagation strategy with cross-entropy as the cost function.

#### 2.2.1. Gaussian kernel optimization for classification

In general, the Gaussian kernel is preferred in pattern classification applications since it aims at finding an RKHS with universal approximating ability, not to mention its mathematical tractability. Nonetheless, to account for the variance of each space when measuring the pairwise distance between samples ***x***_*n*_ and $x_{n^{\prime }}$, the Gaussian kernel relies on the generalized Euclidean metric that is parameterized by a linear projection matrix ***W*** in the form:

(3) $$\kappa_{X}\left(W,\sigma \right)=\exp\left(-\left(x_{n}-x_{n^{\prime }}\right)WW^{⊤}{\left(x_{n}-x_{n^{\prime }}\right)}^{⊤}/2\sigma^{2}\right)$$

where σ ∈ ℝ^+^ is the kernel bandwidth that rules the observation window within the similarity distance is assessed.

In terms of the projection matrix, the formulation of the CKA-based optimizing function in Equation (2) can be integrated into the following kernel-based learning problem:

(4a) $$\begin{array}{l} \hat{W}=\arg\;\underset{W}{\min}\left\{-\log\left(\hat{\rho }\left({\overset{-}{K}}_{X}(W),{\overset{-}{K}}_{L}\right)\right)\right\},\\ \;=\arg\;\underset{W}{\min}\{\log\left(\text{tr}\left(K_{X}(W)\overset{-}{I}K_{L}\overset{-}{I}\right)\right) \end{array}$$

(4b) $$-\frac{1}{2}\log\left(\text{tr}\left(K_{X}(W)\overset{-}{I}K_{X}(W)\overset{-}{I}\right)\right)\},$$

where the logarithm function is used for mathematical convenience. Therefore, the first term in Equation (4b) assesses the similarity between input and target kernels while the second one works as a regularization term minimizing the norm of the input kernel.

#### 2.2.2. Network pretraining for NN classification

From Equation (3), it holds that the projection matrix defines a new space linearly dependent on the input features, that is, ***y*** = ***xW***. Consequently, the generalized Euclidean metric also defines in the new space the following kernel function:

(5) $$\kappa_{y}(y,y^{\prime })=\kappa_{X}(xW,x^{\prime }W)$$

The above metric learning can be read as a particular case of a multi-layer architecture, having a single layer and a linear transfer function. This type of architectures sequentially unravels input data through the stacked linear/non-linear mappings aiming to estimate the output data. Therefore, an *M*-layered Artificial Neural Network (ANN) can be employed to predict the label of an input sample through a battery of feed-forward deterministic transformations, which are implemented by the hidden layers ***h***^*m*^, mapping the input sample *x* to the network output ***h***^*M*^ as below (Bengio, 2009):

(6) $$\{\begin{array}{l} h^{m}=\vartheta (b^{m}+h^{m-1}W^{m}),\forall m=1,\ldots ,M-1\\ h^{0}=x \end{array}$$

where $b^{m}\in {\mathbb{R}}^{Q_{m+1}}$ is the *m*-th offset vector, $W^{m}\in {\mathbb{R}}^{Q_{m+1}\times Q_{m}}$ is the *m*-th linear projection, $Q_{m}\in {\mathbb{Z}}^{+}$ is the size of the *m*-th layer, and ϑ(·) ∈ ℝ is the transfer function that non-linearly maps, saturating each scalar input element. Besides, the first and last layers of the network are always fixed to the input and output dimensions of the learning task, that is, ***h***^0^ ∈ ℝ^*P*^ and ***h***^*M*^ ∈ L⊂{0, 1}^*C*^ with *C* mutually exclusive classes.

It is worth noting that the architectures in Equation (6) attempt to encode into each hidden space the most discriminative attributes of the available dataset, managing the layers. In this line of analysis, we propose a pre-training procedure to provide a suitable initial set of projections {***W***^*m*^}. To this end, the matrix $H^{m}\in {\mathbb{R}}^{N\times Q_{m}}$ is defined to hold every *m*-th non-linear projection of the provided input data ***X***, assuming ***H***^0^ = ***X***. As a result, all pairwise similarities between samples, at *m*-th layer, are gathered into the kernel matrix $K_{m}\in {\mathbb{R}}^{N\times N}$ with elements computed as $\kappa_{m}\left(d\left(h_{n}^{m},h_{n^{\prime }}^{m}\right)\right)$, being $d:{\mathbb{R}}^{Q_{m}}\times {\mathbb{R}}^{Q_{m}}\to {\mathbb{R}}^{+}$ a distance operator defined as follows:

(7) $$d\left(h_{n^{\prime }}^{m},h_{n^{\prime }}^{m}\right)=\left(h_{n}^{m}-h_{n^{\prime }}^{m}\right){\left(h_{n}^{m}-h_{n^{\prime }}^{m}\right)}^{⊤}$$

where every $h_{n}^{m}\in {\mathbb{R}}^{Q_{m}}$ holds each linear projection that we choose of a saturated class, that is, $h_{n}^{m}=\vartheta \left(h_{n}^{m-1}W^{m}\right)$.

With the aim of making our proposal suitable for supervised learning tasks, we introduce the pre-training of ***W***^*m*^ at each layer along with the provided prior knowledge into the CKA optimization framework of Equation (4b). Hence, each matrix ***W***^*m*^ is learned by maximizing the similarity between ***K***_*m*_ and ***K***_*L*_ through the real-valued function that evaluates the alignment between both kernels in Equation (2). Thus, the pre-trained set $\left\{{\hat{W}}^{m}\right\}$ that initializes the network layers provides an assembly of discriminative linear projections, matching the most the distribution of the projected data ***H***^*m*^ and target information *l*.

For both the metric learning and network pre-training, the parameters of the kernel-based learning problem defined in this section are optimized in order solve the problem in Equation (4b). Since, the kernel-based objective is a nonlinear function of the parameters, we use the iterative stochastic gradient method for optimization (Brockmeier et al., 2014). In particular, the CKA optimization procedure is initialized from the principal component mapping of the data, which meets orthogonality conditions. Nonetheless, the span of the resulting projected space is not necessarily orthogonal since there are no restrictions on optimization. Resulting from the optimization, the proposed transformation applies any linear mapping of the data so that the classes are easier to identify. In the case of the metric learning, the projection matrix can be seen as an affine transformation where classes are spatially separated but the nonlinear properties are held. In the case of the pre-training, a nonlinear mapping unravels the data distribution to better classify the samples.

## 3. Experimental set-up

**Figure 1:**
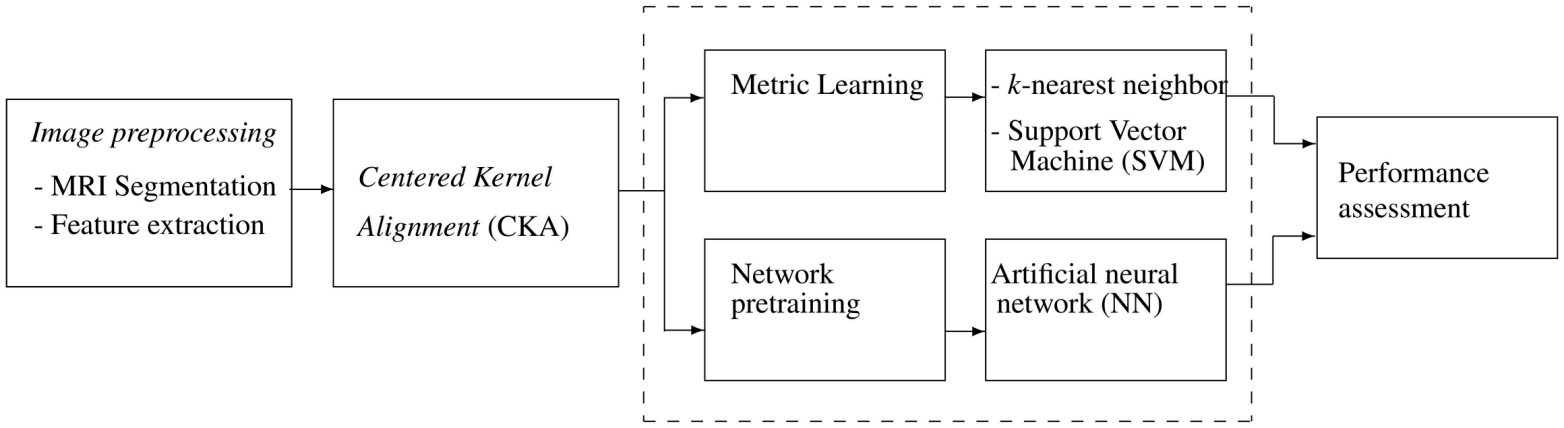
Schematic training pipeline: Input MRI is automatically segmented and a set of features are further extracted. CKA learns a metric or pre-trains network weigths. Tuned classifiers (*k*-nn, SVM, and ANN) are used for computer-aided diagnosis task.

This work introduces a metric learning framework, based on kernel alignment, to support classification tasks of computer-aided diagnosis of dementia. Specifically, validation is accomplished by grouping structural Magnetic Resonance Images (MRI) into one of the following three neurological classes: *Normal Control* (NC), *Mild Cognitive Impairment* (MCI), and *Alzheimer's Disease* (AD). Figure 1 outlines the training pipeline, where the highlighted box contains the proposed data representation stage based on CKA that can be introduced into three considered classification machines.

### 3.1. MRI database and preprocessing

The proposed metric learning is evaluated on a set holding 3,304 MRIs obtained from 896 adult patients selected from the Alzheimer's Disease Neuroimaging Initiative (ADNI) database^1^. This data collection aims at measuring the progression of dementia from serial MRIs, positron emission tomography, other biosignals, and neuropsychological assessments. Table 1 briefly describes the demographic information of the tested cohort of ADNI MRIs, which is split into two subsets. The first one holds 30% of the subjects and is devoted to a blindfolded assessment of the performance framework. The remaining 70% of subjects is employed for framework parameter tuning, which is carried by a 5-fold cross-validation scheme to guarantee that all images of the same subject are assigned to a single group of data analysis (i.e., a validation fold or the test subset).

**Table 1:** Demographic details of the selected ADNI dataset cohort, distributed over the three classes.

	**HC**	**MCI**	**AD**
Patients	231	379	286
*N*	1,048	1,433	823
Age	77.1 ± 5.2	75.9 ± 7.1	76.2 ± 7.2
Male	53.4%	65.2%	54.7%

The tested set of structural MRIs if automatically pre-processed via the widely used FreeSurfer software package^2^ that computes the needed morphological measurements with suitable test-retest reliability across scanner manufacturers and field strengths. Firstly, the bias field of the structural images is corrected as well as its intensity is normalized. Further, the gray and white matter are segmented, and the brain cortex is parcellated. The shapes of resulting structures are further tessellated. Then, structure-wise thickness, area, and volume measurements are computed. The volume features are normalized to the Total Intracranial Volume (Buckner et al., 2004). As a result, an input feature matrix ***X*** with size *N* = 3304 and *P* = 310 is built using the features from each MRI as summarized in Table 2.

**Table 2:** Morphological features extracted by FreeSurfer.

**Type**	***P*_*F*_**	**Units**
Cortical volumes (CV)	69	mm^3^
Subortical volumes (SV)	37	mm^3^
Surface area (SA)	68	mm^2^
Thickness average (TA)	68	mm
Thickness std. (TS)	68	mm
Total feature set	310	

*P_F_, Number of features*.

### 3.2. Parameter setting and network topology

As regards the CKA implementation, the kernel function is computed as below:

(8) $$\begin{array}{l} {\overset{-}{\kappa }}_{Z}\left(z,z^{\prime }\right)=\kappa_{Z}\left(z,z^{\prime }\right)-{𝔼}_{z^{\prime }}\left\{\kappa_{Z}\left(z,z^{\prime }\right)\right\}-{𝔼}_{z}\left\{\kappa_{Z}\left(z,z^{\prime }\right)\right\}\\ \;+{𝔼}_{zz^{\prime }}\left\{\kappa_{Z}\left(z,z^{\prime }\right)\right\},\forall z,z^{\prime }\subset Z \end{array}$$

Therefore, provided a finite sample {(*x*_*n*_, *l*_*n*_):*n* ∈ *N*}, elements ${\overset{-}{k}}_{nn^{\prime }}={\overset{-}{\kappa }}_{Z}\left(z_{n},z_{n^{\prime }}\right)$ in Equation (8) are estimated by the following centered kernel function:

(9) $${\overset{-}{k}}_{nn^{\prime }}=k_{nn^{\prime }}-{𝔼}_{n}\left\{k_{nn^{\prime }}\right\}-{𝔼}_{n^{\prime }}\left\{k_{nn^{\prime }}\right\}+{𝔼}_{n,n^{\prime }}\left\{k_{nn^{\prime }}\right\}.$$

In the validation stage, we consider two scenarios of diagnosis, namely, multi-class task (i.e., MCI, HC, and AD) and three bi-class tasks: HC vs. MCI, HC vs. AD, and MCI vs. AD. In all cases, each compared classifier is adjusted to the following parameter setting:

– *k* Nearest Neighbors (*k*-nn): The number of neighbors is tuned by heuristic searches according to the best performance reached within the specific range [1, 3, 5, 7, 9], resulting in *k* ={7, 9} as the optimal parameter for the baseline and metric learning approaches, respectively.
– Support Vector Machine (SVM): The regularization parameter of the soft margin is searched within the range *C*∈[0.1, 1, 10, 100, 1000]. The SVM-based classifier is trained by the sequential minimal optimization solver for a Gaussian kernel with bandwidth tuned following the algorithm proposed by Álvarez-Meza et al. (2014) that achieves the highest accuracy at *C* = 10 for the baseline and *C*=0.1 for the metric learning approach.
– Neural Networks (NN): Architectures with one and two hidden layers (*M* = {1, 2}) with *m*_1_ = *m*_2_ = *m* are considered for all experiments. Activation functions of the hidden states are the standard sigmoid, ϑ(*z*) = (tanh(*z*) + 1)/2, while at the output layer the following function is selected:
  $$h_{c}^{M}=\exp(b_{c}^{m}+w_{c}^{M}h^{M-1})/\sum_{j}\{\exp(b_{j}^{m}+w_{j}^{M}h^{M-1})\}$$

where $b_{c}^{M}$ is the *c*-th element of ***b***^*M*^, $w_{c}^{M}$ the *c*-th row of ***W***^*M*^, and $\underset{c}{\sum }h_{c}^{M}=1$. Also, we tune the hidden layer size using the exhaustive search strategy for the highest reached accuracy. To this end, the NN classifier is iteratively trained through a range of log-spaced layer sizes bounded by *m* ∈ [*C, P*−1]. The exhaustive search for the layer size attained *m*_1_ = 11 for 1-layered network, either randomly initialized or with supervised pre-training. The 2-layered architecture obtains the highest accuracy at *m*_1_ = *m*_2_ = 11 when randomly initialized and *m*_1_ = *m*_2_ = 5 when pre-trained.

The diagnosis performance is assessed by the classification accuracy *a* and true positive fraction for the corresponding class (τ_*c*_), defined as follows:

(10a) $$a={𝔼}_{c}\left\{t_{c}^{p}+t_{c}^{n}\right\}$$

(10b) $$\tau_{c}=\;t_{c}/N_{c},\;c=\{MCI,HC,AD\}$$

being $t_{c}^{p}$, $t_{c}^{n}$, and *N*_*c*_ the number of true positives, true negatives, and total samples for *c*-th class, respectively. Note that the true positive fractions provide information about the sensitivity and the specificity. Thus, for the multi-class case, τ_*AD*_ and τ_*MCI*_ can be interpreted as the two-class sensitivity, while τ_*NC*_ agrees with the two-class specificity (Bron et al., 2015).

## 4. Results and discussion

For enhancing the computer-aided diagnosis of dementia, we explore a metric learning framework based on the centered kernel alignment function, aiming to estimate more discriminative spaces. Evaluation prove the benefits of the proposed CKA-based meatric learning framework to a certain degree. From the obtained experimental results the following considerations are to be taken into account:

The use of a kernel learning method, termed Centered Kernel Alignment, allows quantifying the similarity between the input sample space and corresponding class label set. However, the core problem of implementing CKA is the way of choosing an appropriate kernel and setting its parameters, tending to guarantee good generalization performance of the learning machine. Besides its generalization ability, the Gaussian kernel is employed due to the possibility to incorporate a more generalized Euclidean metric that accounts for the variance of the feature and label spaces through a projection matrix. Therefore, the applied supervised metric learning projects the feature set so that the CKA value becomes maximal. However, the alignment optimization procedure is strongly dependent on the metric model. That is, simple linear mappings are easier to tune but cannot encode higher order relations. On the other hand, multi-layer architectures can discover data non-linearities, but are more expensive to tune.

The CKA dependence using the Mahalanobis metric learning is developed for three commonly used approaches to supervised classification: *k*-nn and SVM that are fed into a gradient-based optimization, and NN that are optimized using a backpropagation strategy with cross-entropy as the cost function. For the NN classifier, we generalize the supervised linear projection as a multilayer neural network, having one layer provided with a linear transfer function. Thus, a supervised CKA-based network pre-training is developed by applying the CKA procedure sequentially from the input to the output layer.

A bootstrapping procedure on the test set with 1,000 resamples estimates the average and the 95% confidence interval (CI) of each performance measure, as suggested by Bron et al. (2015). Besides, a two-sample *t*-test assesses the significative difference between a classifier and its CKA-enhanced version at a significance level of (1%). From the obtained results, it follows that the either case of the CKA metric learning gives rise to the classification performance for the multi-class scenario. Table 3 displays the validation performance of the multi-class scenario presented by each classifier. Due to the assumed influence of the topology, we also consider two versions of NN: 1-layered and 2-layered. In the first case of classification (top four rows), when feeding just the input feature set, the 2-layered NN classifier produces the best performance (average 57.4%), while the baseline *k*-NN certainly yields the worst results (45.5%). The bottom four rows hold the results of using the suggested learned metric, showing that the performance improves for most of classifiers. The last column computes the standard deviation of the true-positive rates (*std*_τ_) a bias measure. As seen, the SVM is the most balanced classifier and benefits from the CKA by decreasing its standard deviation (*std*_τ_ = 4.2%). The gray-shaded cells denote the best performance of each classifier accuracy for all cases, noting that the pre-trained 1-layered NN is the best configuration. Another aspect to remark is that the CKA-based metric learning improves every one of the performance measures regardless of the used classifier as shown in the row *Performance after CKA*.

**Table 3:** Testing performance of the metric learning for the multi-class task.

	***a (CI)***	**τ_*HC*_*(CI)***	**τ_*MCI*_*(CI)***	**τ_AD_*(CI)***	***std*_τ_(%)**
*k*-nn	45.5 (42.2–48.4)	51.9 (46.6–57.3)	45.5 (40.9–50.6)	37.3 (31.1–43.5)	7.3
SVM	53.5 (50.4–56.4)	56.2 (50.8–61.8)	48.2 (43.4–52.8)	59.5 (53.1–65.4)	5.8
1-layer NN	57.0 (53.6–59.7)	58.3 (52.6–63.6)	61.3 (56.4–65.8)	47.6 (41.4–53.7)	7.2
2-layer NN	57.4 (54.6–60.4)	69.8 (64.5–74.6)	42.6 (38.1–47.1)	67.5 (61.3–73.1)	15.1
Perf. before CKA	53.4	59.1	49.4	53.0	
ML + *k*-nn	53.8 (50.7–56.7)^*^	63.9 (58.2–69.3)^*^	50.9 (46.2–55.3)^*^	46.0 (40.3–52.2)^*^	9.3
ML + SVM	57.7 (54.4–60.7)^*^	62.7 (57.5–67.8)^*^	54.7 (50.2–59.6)^*^	56.7 (50.6–62.4)	4.2
Pretrained 1-layer NN	60.1 (56.9–62.7)^*^	66.0 (60.2–70.9)^*^	59.2 (54.7–64.0)	54.0 (48.1-59.8)^*^	6.0
Pretrained 2-layer NN	58.5 (55.4–61.5)^*^	72.2 (69.1–76.9)^*^	44.1 (39.4–48.6)^*^	66.3 (60.6-71.9)	14.8
Perf. after CKA	57.5	66.2	52.2	55.8	

*Average and confidence interval (CI) for 1,000 resamples bootstrapping are reported*.

* *Indicates whether the performance after CKA is larger than before CKA for each classifier at a significance level of 1%*.

*The gray shade denotes the best result for each performance measure*.

Likewise, the use of CKA also enhances the classification of each considered bi-class task as shown in Table 4. The distinction between HC and AD classes presents the highest values of performance (above 92% in average), meaning that this task is likely to have the easiest discriminating representation. However, the more challenging the bi-class task, the lower the increasing of classification performance.

**Table 4:** Testing performance of the metric learning for the bi-class task.

	***a (CI)***	**τ_*HC*_*(CI)***	**τ_*MCI*_*(CI)***	**τ_*AD*_*(CI)***	***std*_τ_ (%)**
**HC vs. AD**				
*k*-nn	80.7 (77.5–83.7)	94.8 (91.7–96.6)	NA	61.8 (55.5–67.5)	23.3
SVM	90.6 (88.0–92.7)	88.9 (84.8–92.0)	NA	92.9 (88.6–95.5)	2.8
1-layer NN	91.2 (88.3–93.1)	95.7 (93.3–97.6)	NA	85.1 (79.7–89.0)	7.5
2-layer NN	91.9 (89.2–93.6)	94.4 (91.6–96.5)	NA	88.4 (83.9–92.1)	4.2
Perf. before CKA	88.6	93.5	NA	82.1	
ML + *k*-nn	90.4 (87.4–92.6)^*^	96.9 (94.6–98.4)^*^	NA	81.7 (75.8–86.3)^*^	10.7
ML + SVM	91.5 (88.7–93.5)	93.2 (90.1–95.6)^*^	NA	89.2 (84.0–92.7)^*^	2.8
Pretrained 1-layer NN	92.0 (89.4–93.8)	95.1 (92.4–97.2)	NA	88.0 (83.0–91.6)^*^	5.0
Pretrained 2-layer NN	92.6 (89.7–94.2)	95.7 (92.9–97.5)	NA	88.4 (83.5–91.8)	5.2
Perf. after CKA	91.6	95.2	NA	86.8	
**HC vs. MCI**					
*k*-nn	59.5 (55.7–62.7)	51.1 (45.8–56.3)	65.9 (61.2–70.0)	NA	10.5
SVM	64.9 (61.3–68.3)	40.6 (35.0–45.9)	83.5 (79.6–86.8)	NA	30.3
1-layer NN	70.4 (67.3–73.3)	59.7 (54.1–64.9)	78.6 (74.3–81.9)^*^	NA	13.4
2-layer NN	71.9 (68.5–74.8)	59.1 (53.6–64.0)	81.6 (77.6–84.7)	NA	15.9
Perf. before CKA	66.7	52.6	77.4	NA	
ML + *k*-nn	64.7 (60.8–67.9)^*^	66.5 (61.0–71.4)^*^	63.3 (58.4–67.8)^*^	NA	2.3
ML + SVM	66.0 (62.3–69.1)	59.4 (53.7–64.7)^*^	71.1 (66.3–75.1)^*^	NA	8.3
Pretrained 1-layer NN	72.5 (69.1–75.6)^*^	61.2 (56.1–66.5)	81.2 (77.3–89.8)^*^	NA	14.1
Pretrained 2-layer NN	71.2 (68.0–74.0)	57.2 (51.5–62.0)	86.9 (78.1–85.3)	NA	21.0
Perf. after CKA	68.6	61.1	74.4	NA
**MCI vs. AD**					
*k*-nn	67.0 (63.3–70.8)	NA	84.4 (80.6–87.5)	39.5 (33.7–46.2)	31.7
SVM	68.5 (64.7–72.0)	NA	76.0 (71.8–80.3)	56.6 (50.8–63.0)	13.7
1-layer NN	67.9 (63.8–71.1)	NA	74.3 (70.0–78.4)	57.8 (51.6–63.6)	11.7
2-layer NN	71.1 (67.3–74.1)	NA	79.0 (75.2–82.8)	58.6 (52.0–64.7)	14.4
Perf. before CKA	68.6	NA	78.4	53.1	
ML + *k*-nn	68.5 (65.0–71.8)^*^	NA	78.7 (74.4–82.5)^*^	52.3 (46.8–58.8)^*^	18.7
ML + SVM	69.4 (65.3–72.6)	NA	78.2 (74.1–82.2)	55.5 (49.5–61.3)	16.1
Pretrained 1-layer NN	69.2 (65.5–72.6)	NA	78.7 (74.7–82.4)^*^	54.3 (48.5–60.3)^*^	17.3
Pretrained 2-layer NN	72.4 (69.1–75.8)	NA	85.4 (81.6–88.5)^*^	52.0 (46.5–58.8)^*^	23.6
Perf. after CKA	69.9	NA	80.3	53.5	

*Average and confidence interval (CI) for 1,000 resamples bootstrapping are reported*.

* *Indicates whether the performance after CKA is larger than before CKA for each classifier at a significance level of 1%*.

*The gray shade denotes the best result for each performance measure*.

A detailed analysis of each performance measure shows that the developed metric increases the accuracy obtained by all classification machines for each bi-class task. Also, the proposed metric increases the percentage of true positive fractions achieved by most of classifiers for each neurological class, improving the sensitivity of pathological groups and the specificity of healthy controls, especially for the HC vs. AD and HC vs. MCI task. Specifically, for the HC vs. MCI bi-class task, the proposed metric learning enhances significantly the performance balance of the *k*-nn and SVM classifier (2.3 and 8.3%, respectively) in comparison with their performance without ML (10.5 and 30.3%, respectively). Therefore, CKA forces each hidden space to increase the class separability by sequentially increasing the match between input and target data distributions. Nonetheless, most of the classifiers underperform for the MCI class due to its heterogeneous distribution. This situation arises because MCI is an intermediate disease between HC and AD, so that some patients may eventually convert to any of the other two classes.

For the sake of comparison, the proposed methodology of training is contrasted against the classification results for CADDementia challenge of AD diagnosis in Bron et al. (2015). To this end, the accuracy, true-positive rate, and area under the ROC (Receiver operating curve) are recomputed following the same evaluation scheme above described. Tables 4, 5 report the obtained results of CKA-enhanced SVM and NN machines along with the best performing approach in the challenge for each measure. It is worth noting that we can contrast results because class distributions and data acquisition protocols are similar for both works. However, we make clear that the comparison is given only in terms of the average and confidence intervals since the hypothesis test can not be performed.

**Table 5:** Accuracy performance measures for the multi-class scenario following the validation scheme in Bron et al. (2015).

	***a (CI)***	**τ_*HC*_*(CI)***	**τ_*MCI*_*(CI)***	**τ_*AD*_*(CI)***	***std*_τ_ (%)**
ML + SVM	57.7 (54.4–60.7)	62.7 (57.5–67.8)	54.7 (50.2–59.6)	56.7 (50.6–62.4)	4.2
Pretrained 1-layer NN	60.1 (56.9–62.7)	66.0 (60.2–70.9)	59.2 (54.7–64.0)	54.0 (48.1–59.8)	6.0
Pretrained 2-layer NN	58.5 (55.4–61.5)	72.2 (69.1–76.9)	44.1 (39.4–48.6)	66.3 (60.6–71.9)	14.8
Soerensen-equal	63.0 (57.9–67.5)	96.9 (92.9–99.2)	28.7 (21.3–37.4)	61.2 (51.6-69.8)	34.11
Abdulkadir	53.7 (48.3–58.2)	45.7 (37.0–53.6)	65.6 (56.1–73.0)	49.5 (39.4–58.8)	10.6
Routier-train	48.3 (42.9–53.4)	48.1 (39.8–56.9)	21.3 (14.8–29.0)	80.6 (72.2–87.3)	29.7
Ledig-ALL	57.9 (52.5–62.7)	89.1 (83.7–93.8)	41.0 (32.4–49.6)	38.8 (30.7–50.0)	28.4
Wachinger-step1	54.0 (48.9–59.0)	68.2 (60.2–75.4)	41.0 (31.9–50.9)	51.5 (42.2–61.1)	13.7

*The gray shade denotes the best result for each performance measure*.

Results in Table 5 show that no approach achieves the best performance in all measures. In the case of the accuracy, Soerensen-equal outperforms with *a* = 63.0%, followed by the CKA-enhanced SVM and NN machines. Regarding the true-positive rates, Soerensen-equal achieves the highest τ_*NC*_ = 96.9 at the cost of decreasing τ_*MCI*_ and τ_*AD*_ (e.g., τ_*AD*_ = 61.2). Similarly, Abdulkadir and Routier-train algorithms bias toward AD (τ_*AD*_ = 80.6) and MCI (τ_*MCI*_ = 65.6), so that they perform the worst in MCI (τ_*MCI*_ = 21.3) and HC (τ_*HC*_ = 45.7), respectively. Therefore, the within approach true-positive rates, that vary largely, prove that some algorithms incline toward one class but reducing the discrimination of the other classes. In contrast, the proposed CKA enhancement provides more similar τ and a more balanced performance. Such a fact is proved by the standard deviation of the true-positives (*std*_τ_), where the enhanced SVM and pre-trained 1-layered NN vary the least in comparison with results reported in the CADDementia challenge.

From Table 6 it follows that the per-class AUCs also depends on the selected approach. For example, Ledig-All and Wachinger-step1 algorithms provide the best AUC for HC and AD class, respectively. However, the former achieves 59.7% for MCI and the latter becomes the worst-performing. On the contrary, the proposed approach, particularly the pre-trained NN, achieve the best AUC for the MCI with no compromise of the other two classes. These results prove that the CKA criterion aims at discriminating all classes simultaneously obtaining balanced results and avoiding class biasing.

**Table 6:** Area under the ROC performance measures for the multi-class scenario following the validation scheme in Bron et al. (2015).

	***AUC* (CI)**	***AUC*_*HC*_ (CI)**	***AUC*_*MCI*_ (CI)**	***AUC*_*AD*_ (CI)**	***std*_*AUC*_ (%)**
ML + SVM	74.7 (72.1–77.0)	77.6 (74.2–80.7)	61.3 (57.6–64.9)	82.3 (78.7–84.9)	11.0
Pretrained 1-layer NN	78.5 (76.3–80.3)	82.4 (79.8–84.9)	64.5 (61.0–67.7)	85.2 (82.5–87.5)	11.2
Pretrained 2-layer NN	77.9 (75.8–79.8)	81.5 (78.5–83.9)	62.7 (59.1–65.9)	85.5 (82.9–87.9)	12.2
Soerensen-equal	78.8 (75.6–82.0)	86.3 (81.8–89.3)	63.1 (56.6–68.3)	87.5 (83.4–91.1)	13.8
Abdulkadir	77.7 (74.2–81.0)	85.6 (81.4–89.0)	59.9 (54.1–66.4)	86.7 (82.3–90.3)	15.2
Ledig-ALL	76.7 (73.6–79.8)	86.6 (82.7–89.8)	59.7 (53.3–65.1)	84.9 (79.7–88.7)	15.1
Wachinger-step1	74.6 (70.8–78.1)	79.1 (73.5–83.1)	55.0 (48.5–61.4)	89.2 (85.3–92.3)	17.6

*The gray shade denotes the best result for each performance measure*.

## 5. Conclusion

This work introduces a supervised metric learning to support MRI classification. The proposed learning decodes discriminant information based on the maximization of the similarity between the input distribution and the corresponding target (diagnosis classes), aiming at enhancing the class separability. Thus, the conventional centered kernel alignment (CKA) is introduced as the cost function to infer the metric paramters that feed two classifiers (*k*-nn and SVM) and initialize the training of an NN. Evaluation of the proposed metric learning framework is carried out on the well-known ADNI dataset, where several morphological measurements are extracted using FreeSurfer to represent each MRI scan. In order to avoid over-fitting and prevent a biased classification performance, we split the dataset into two groups: A training subset for parameter tuning, and a test subset for performance assessment. Experiments are conducted according two diagnosis scenarios. For the multi-class one, our proposed CKA improves the performance for all classifiers in terms of the classification accuracy and the true positive fraction of each neurological class. In particular, the 1-layered NN classifier achieves the best performance (average 57.3%), and *k*-nn reaches competitive results (46.4%). In the bi-class scenario, CKA also enhances the classification, attaining measures over 90% in average for HC vs. AD task. Therefore, introducing CKA in the supervised metric learning determines spaces with a better class separability that increases both the sensitivity and specificity. As an additional benefit, the proposal better balances the performance in each class.

Attained results in the work yield the following research directions: Since the span of the resulting space is not guaranteed to be orthogonal, CKA criterion may produce redundant information. Then we propose to further introduce orthogonality constraints between the bases to solve this issue. Also, because the considered input feature set corresponds to a standard biomarker extraction, it is interesting to analyze other kinds of image representation strategies aiming at finding their relevance for class discrimination assessed by the CKA criterion. Besides, we devise two improvements of the sequential pre-training, namely, an NN training using CKA as the cost function and the evaluation of other multi-layer machines as Generative Stochastic Networks. Finally, we note that the class-wise performance can be parameterized by the introduced kernel function in the target space, so that a larger similarity of a particular class should increase its true positive rate.

## Author contributions

All authors listed, have made substantial, direct and intellectual contribution to the work, and approved it for publication.

### Conflict of interest statement

The authors declare that the research was conducted in the absence of any commercial or financial relationships that could be construed as a potential conflict of interest.

## Footnotes

^1^www.adni-info.org

^2^freesurfer.nmr.mgh.harvard.edu
